# Ultrafast Q-boosting in semiconductor metasurfaces

**DOI:** 10.1515/nanoph-2023-0718

**Published:** 2024-02-19

**Authors:** Ziwei Yang, Mingkai Liu, Daria Smirnova, Andrei Komar, Maxim Shcherbakov, Thomas Pertsch, Dragomir Neshev

**Affiliations:** ARC Centre of Excellence for Transformative Meta-Optical Systems (TMOS), Department of Electronics Materials Engineering, Research School of Physics, 2219 Australian National University, Canberra, ACT 2600, Australia; Institute of Applied Physics, Abbe Center of Photonics, Friedrich Schiller University Jena, Albert-Einstein-Straße 15, 07745 Jena, Germany; Department of Electrical Engineering and Computer Science, University of California, Irvine, CA 92697, USA

**Keywords:** all-optical modulation, direct bandgap semiconductors, time-variant metasurfaces

## Abstract

All-optical tunability of semiconductor metasurfaces offers unique opportunities for novel time-varying effects, including frequency conversion and light trapping. However, the all-optical processes often induce optical absorption that fundamentally limits the possible dynamic increase of their quality factor (Q-boosting). Here, we propose and numerically demonstrate the concept of large Q-boosting in a single-material metasurface by dynamically reducing its structural anisotropy on a femtosecond timescale. This balance is achieved by excitation with a structured pump and takes advantage of the band-filling effect in a GaAs direct-bandgap semiconductor to eliminate the free-carrier-induced loss. We show that this approach allows a dynamic boosting of the resonance quality factor over orders of magnitude, only limited by the free-carrier relaxation processes. The proposed approach offers complete dynamic control over the resonance bandwidth and opens applications in frequency conversion and light trapping.

## 1 Introduction

Over the last decade, optical metasurfaces have developed into a versatile platform for the generation, manipulation, and detection of engineered light wavefronts [1], [2], [3]. In addition to their unique optical properties, the nanoscale volume of their constituent meta-atoms opens exciting opportunities for efficient tuning of their functionalities in real-time, thereby enabling new dynamically reconfigurable optical devices and systems [4], [5], [6], [7]. The metasurface tunability can be remarkably enhanced by utilizing high-quality (high-Q) factor optical resonances, such as nonlocal [8] and quasi-bound state in the continuum (quasi-BIC) [9], [10] resonances. Importantly, quasi-BIC metasurfaces offer unique opportunities for designing the resonance bandwidth through precision engineering of the structural or permittivity asymmetry [9], [10], [11]. Several different tuning mechanisms have been widely explored, including mechanical [12], thermal [13], and electrical [14], [15] tuning.

Notably, tuning the resonances of quasi-BIC metasurfaces with small stimuli opens the opportunity for all-optical tunability at ultrafast femtosecond timescales. Under such tuning speeds, significant energy of an optical pulse will remain in the metasurface resonators during the tuning process, thereby enabling efficient frequency conversion and non-reciprocal effects [16], [17]. Such ultrafast tunability of metasurfaces facilitates *time-variant effects* and offers new physical regimes of light propagation in dynamic dispersive media [18]. In particular, time-varying quasi-BICs can bypass the bandwidth limitations of optical metasurfaces and enable dynamic capture of light pulses [19]. For example, by dynamically increasing the Q-factor of the resonances, called Q-boosting, one can capture broadband pulses and translate them with high efficiency into a new frequency-shifted state.

The all-optical ultrafast tuning is enabled by various nonlinear processes when high-energy laser pulses excite the metasurface. These include single and two-photon absorption, free-carrier generation, and Kerr effects [20], [21]. Several works have explored ultra-fast tunability of metasurfaces fabricated from indirect [20], [22], [23], [24] and direct band-gap [25], [26], [27] semiconductors. Direct bandgap semiconductors offer exceptionally efficient ultrafast free-carrier generation and strong ultrafast modulation. However, the induced free carriers inevitably increase the materials’ optical absorption and dampen the metasurface resonance [28], [29]. This optically-induced damping fundamentally limits the Q-boosting effect and prohibits light trapping [19]. Therefore, practical solutions are needed to enable time-variant metasurfaces with large Q-boosting and strong light-trapping.

Here, we propose and numerically demonstrate the concept of large Q-boosting in a single material quasi-BIC metasurface excited by a structured pump. We utilize the band-filling effect in a GaAs direct-bandgap semiconductor to eliminate the free-carrier-induced absorption and achieve full conversion of a quasi-BIC to a complete BIC mode. To elucidate this effect, we develop a theoretical model that describes the balance between the structural and dynamic asymmetry of a quasi-BIC metasurface and confirm our model by rigorous numerical simulations. We note that such balance cannot be obtained in dual-material metasurfaces [30] as the band-filling effect of the two materials cannot be fully balanced, and only partial Q-boosting can be achieved. Our results open new opportunities for dynamic programming of the Q-factor of metasurfaces with applications in light frequency conversion and trapping.

## 2 Results

### 2.1 Q-boosting by structured pumping

**Figure 1:**
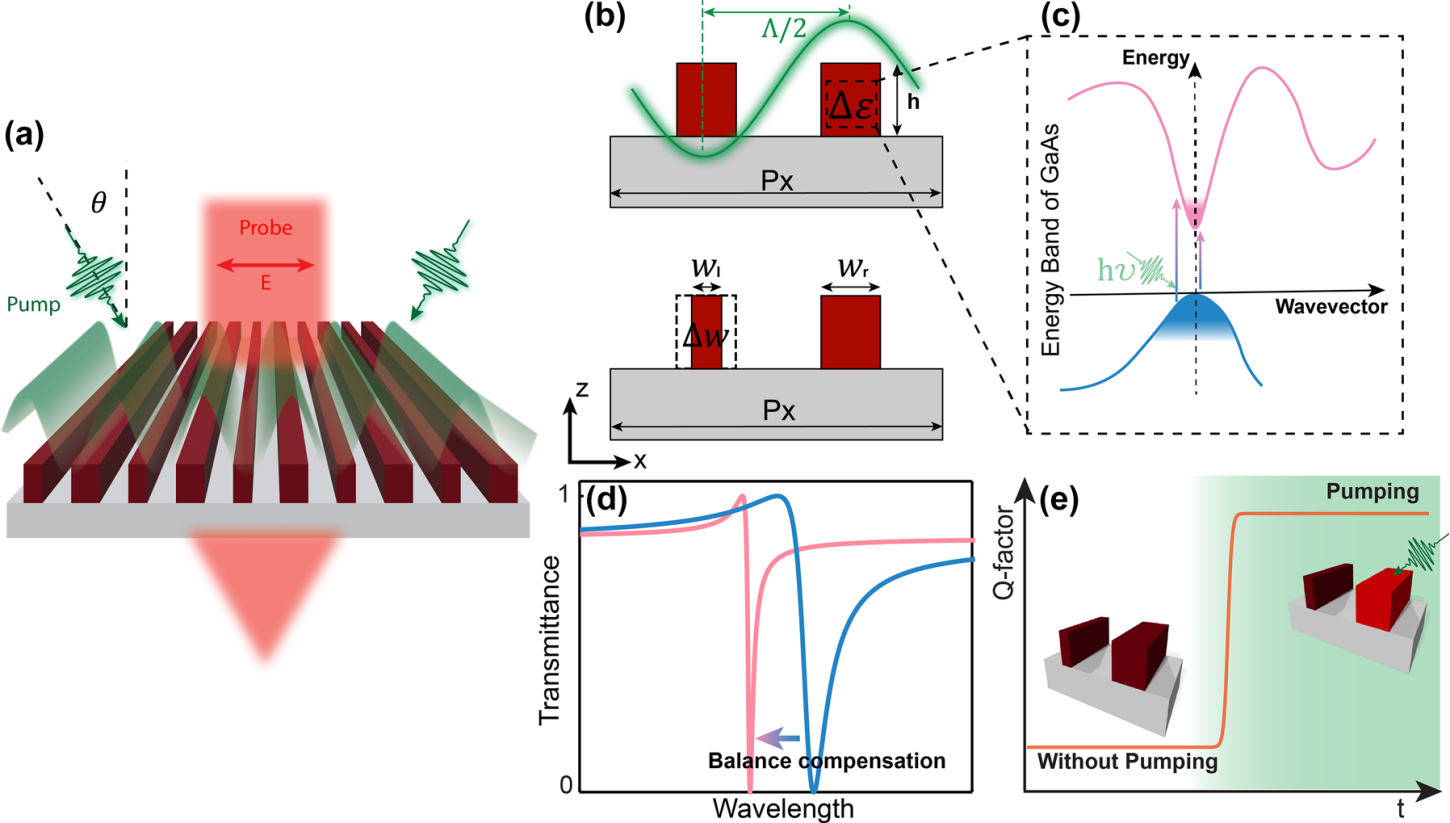
Design of the Q-boosting time-varying metasurface. (a) Schematic of engineering the resonance bandwidth by structured pumping and a homogeneous probe. (b) Side view (*x* − *z* plane) of the unit cell of the binary metasurface. The top panel illustrates the metasurface permittivity disbalance between the two nano-bars, induced by a structured pump from the interference of two beams, green line. The bottom panel represents the geometrical disbalance due to the different widths of the two nano-bars. (c) Free carrier excitation in the GaAs when the pump photon energy is higher than its bandgap. (d) Due to the pumping, there is induced negative permittivity change leading to the resonance shifts from the static transmittance spectrum (blue curve) to a new high-Q state (pink curve) with a narrow bandwidth. This bandwidth manipulation effect can be depicted as a change of the Q-factor versus time, schematically shown in (e).

The concept behind the all-optical manipulation of the resonant bandwidth of a direct bandgap semiconductor metasurface at femtosecond time scales is illustrated in Figure 1(a). The metasurface has a quasi-one-dimensional (1D) geometry and is excited by a structured pump. The metasurface represents a subwavelength grating, which we will refer to as a binary metagrating. The ultrafast modulation of the metasurface enables the balance between the structural and dynamic asymmetry of this metagrating. As a result, one can capture a broadband laser pulse by dynamically converting its wide bandwidth into a narrow-band resonance.

To achieve the ultrafast modulation, we consider a metagrating composed of GaAs semiconductor with a direct bandgap of 1.42 eV (873 nm). The design is based on a thin GaAs layer on a transparent SiO_2_ substrate, following an established fabrication technique [31]. This geometry allows for operation in transmission, which is not possible in conventional GaAs structures based on growth and oxidation process [32]. We employ a period-doubling by reducing the width of one of the nano-bars of the grating to create a supercell, as shown in Figure 1(b). The reduced width of the left nano-bar breaks the in-plane *C*_2_ symmetry and opens a radiation channel from the quasi-BIC mode to free space. Such quasi-BIC mode originates from an infinite Q-factor symmetry-protected BIC mode by symmetry breaking. The Q-factor of the quasi-BIC is inversely proportional to the residual radiative loss [10]. Accordingly, we can engineer the resonance bandwidth by adjusting the structural asymmetry, Δ*w* = *w*_*r*_ − *w*_*l*_, as shown in the bottom panel of Figure 1(b).

An alternative approach to breaking the metagrating symmetry is to change the permittivity of one of the nano-bars by applying a spatially-structured external pumping. Such structured optical pumping can be created by the interference of two laser beams impinging on the metagrating at an angle *θ*, Figure 1(a). This angle *θ* is defined as $\theta =\arcsin\frac{\lambda_{P}}{2\Lambda }$ , where *λ*_*P*_ is the pump laser wavelength. Λ is the periodicity of the interference pattern, which needs to match the periodicity of the binary meta-grating Λ = *P*_*x*_, see Figure 1(a) and (b). As the pump wavelength is significantly shorter than the probe wavelength, the interference pattern can be generated with moderately inclined pump beams. For example, for a pump of *λ*_*P*_ = 532 nm, the half angle *θ*, Figure 1(a), is 29°. Notably, diffractive optical elements can be used to robustly generate such an interference pattern. As a result, only one nano-bar per unit cell is being optically excited.

Upon stimulation of the right nano-bar, as shown in the top panel of Figure 1(b), the electrons will transit from the valence band to the conduction band if the pumping photon energy exceeds the bandgap of the GaAs. When the low energy states in the conduction band are filled, the electrons from the valence band require energies larger than the normal bandgap to populate the high energy states of the conduction band. Hence, the absorption coefficient of the GaAs decreases at energies above the bandgap. This effect is referred to as the band-filling effect and is schematically depicted in Figure 1(c). The pumping process will, therefore, generate a permittivity disbalance between the two nano-bars, denoted as Δ*ɛ* = *ɛ*_*o*_ − *ɛ*_*p*_. Here, *ɛ*_*o*_ is the original permittivity of the GaAs, and *ɛ*_*p*_ is the permittivity under pumping, as shown in Figure 1(b).

Because both geometric asymmetry and permittivity disbalance can modulate the Q-factor of the quasi-BIC mode, the dynamic permittivity disbalance can be harnessed as a means to counterbalance the static geometric asymmetry of the metagrating. Such balance can result in a dynamic spectral narrowing of the metasurface resonance, shown in Figure 1(d). Here, the initially broad bandwidth resonance (blue curve) will be squeezed and become narrower (pink curve) after the pulse pumping. Note the polarization of the probe light is perpendicular to the grating direction, Figure 1(a). We also note that the possible damping due to the excitation of free careers in the GaAs is canceled by band-filling effects when the probe beam has photon energy comparable with the GaAs bandgap.

Notably, the Q-factor of the metasurface resonance undergoes a rapid increase during the pumping process, as shown in Figure 1(e). This rapidly increasing Q-factor happens at a femtosecond timescale shorter than the photon lifetime of the resonator and is thereby termed Q-boosting. In Figure 1(e), the orange curve depicts a step-like boost to the Q-factor at the boundary of pumping. Here, the white background schematically indicates the unpumped state of the metasurface, while the green background indicates its structured-pumped state.

In what follows, we present a generalized method for analyzing the interplay between the geometric and permittivity-based disbalance of the quasi-BIC mode. Subsequently, we will describe the bandwidth engineering of the resonance utilizing an experimentally realistic set of material parameters and permittivity change. Finally, we will apply this femtosecond time-varying regime to demonstrate frequency conversion and energy storage by the metasurface.

### 2.2 Theoretical modeling

**Figure 2:**
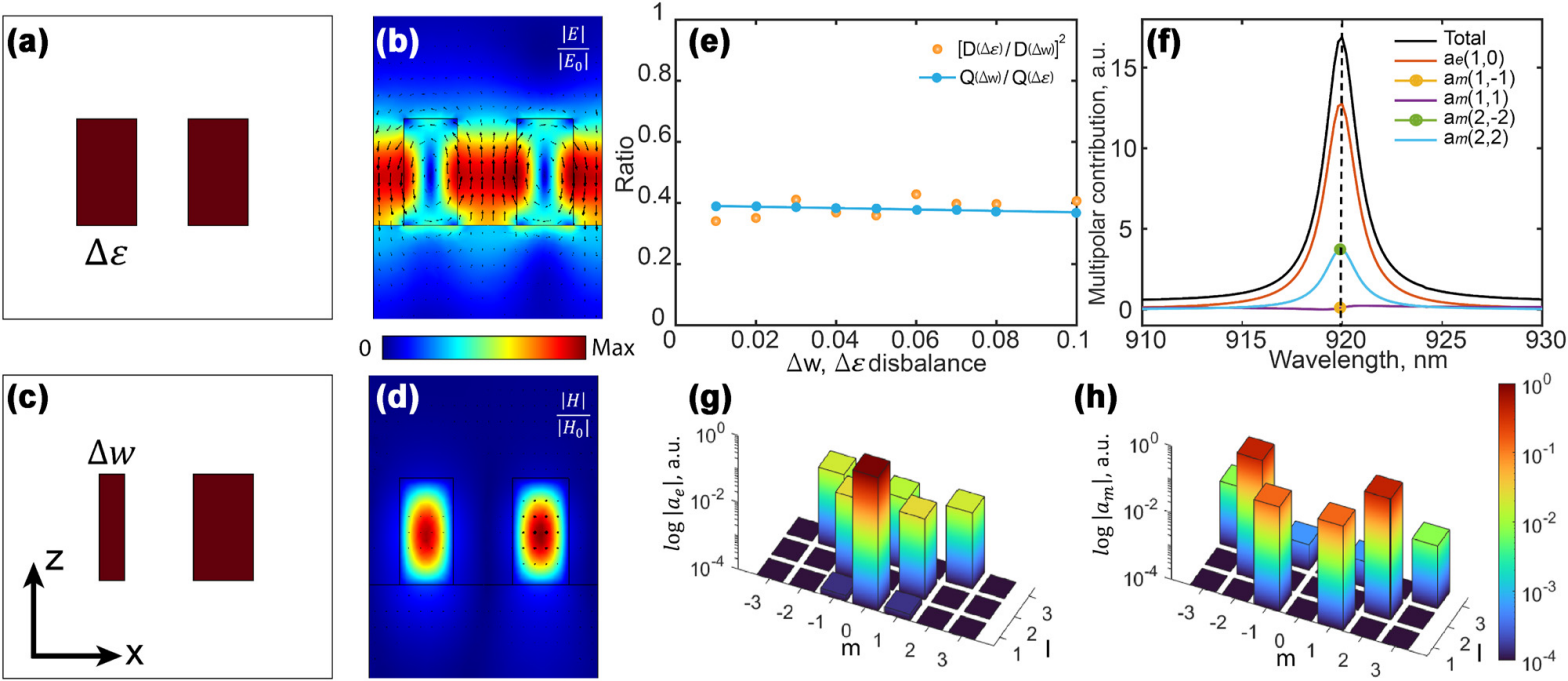
Permittivity vs. geometric metasurface disbalance. Schematics of the metagrating unit cell, viewed in the *x* − *z* plane: (a) nano-bars are of the same width but have slightly different permittivities, and (c) nano-bars have different widths without the permittivity disbalance. (b, d) Normalized magnitudes of the (b) electric and (d) magnetic fields in the quasi-BIC mode at *α*_*w*_ = 0.05. The blue line in (e) shows the ratio of the Q-factors for the same relative change in permittivity (*α*_*ɛ*_) and nano-bar width (*α*_*w*_). That ratio is equivalent to the inverse square ratio of coupling amplitude *D* in Δ*ɛ* and Δ*w* by the multipolar coefficient superposition with the orange circles. (f) Dominant multipoles in the multipolar decomposition of the electromagnetic field in the unit cell near the quasi-BIC resonance. (g, h) 3D log scale bar-plot visualization of the electric |*a*_*e*_| and magnetic |*a*_*m*_| multipolar coefficients for the quasi-BIC mode at *α*_*w*_ = 0.05.

To comprehend the compensation mechanism between the geometric asymmetry and the optically-induced dynamic permittivity disbalance, we first examine two scenarios where the geometric asymmetry (Δ*w*) and permittivity deviation (Δ*ɛ*) originate from the left nano-bar of the metagrating. We assume lossless GaAs with a fixed permittivity *ɛ* = 12.25 and no substrate to completely eliminate the bi-anisotropy due to the symmetry breaking in the *z*-direction. The schematics of a bipartite grating with geometric and permittivity disbalances are depicted in Figure 2(a)–(c). The metagrating is originally designed to support the BIC mode at the wavelength of 920 nm, with a structural period of *P*_*x*_ = 550 nm and a thickness of *h* = 310 nm. In this case, both nano-bars have the same width of *w* = 135 nm and are equally spaced. The symmetry-protected BIC mode does not radiate to the far field, exhibiting an infinitely high Q-factor, as confirmed by the eigenmode calculations conducted using the finite element method (FEM) in COMSOL Multiphysics.

We next investigate the effects of structural asymmetry introduced by the width and permittivity deviations. The asymmetry facilitates the mode leakage into the far field, transforming the nonradiative mode into the quasi-BIC state. An exemplary profile of the quasi-BIC field distribution is depicted in cross-sections in Figure 2(b)–(d). The field vectors in both nano-bars are oriented in opposite directions, diminishing the mode overlap with the outgoing plane waves. To establish a quantitative equivalence between the two symmetry-breaking mechanisms, we define the ratio of the finite quality factors *Q* determined by Δ*w* and Δ*ɛ*,

(1) $$\frac{Q\left(\Delta w\right)}{Q\left(\Delta \varepsilon \right)}={\left[\frac{D_{x}\left(\Delta \varepsilon \right)}{D_{x}\left(\Delta w\right)}\right]}^{2}.$$

For our mode, these are related to the far-field coupling amplitudes *D*_*x*_ of the *x*-polarized radiation channel in the coupled-mode-theory formalism. As discussed in Ref. [10], the coupling coefficient can be expressed through the multipolar moments,

(2) $$D_{x}\propto \left[p_{x}\mp \frac{m_{y}}{c}+\frac{ik_{0}}{6}Q_{zx}^{e}+\cdots \;\right],$$

with ***p*** (electric dipole), ***m*** (magnetic dipole), ***Q*** ^*e*^ (electric quadrupole) being the leading terms of the multipolar expansion. The geometric and permittivity asymmetry can be quantified by the ratio of the deviations with respect to the original values, *α*_*w*_ = |Δ*w*|/*w*_0_ and *α*_*ɛ*_ = |Δ*ɛ*|/*ɛ*_0_. Previous works estimated only the relationship between the induced electric dipole moment and *α*_*w*_ or *α*_*ɛ*_ by using a Taylor expansion in the perturbative regime [11], valid only as an approximation for small *α*_*w*,*ɛ*_ → 0. Here, we take a different approach and calculate *D*_*x*_ up to the quadrupolar-order corrections based on the multipolar decomposition of the numerically obtained eigenmode field distribution in the unit cell (see section S1 of the Supplementary).

The results of the multipolar analysis for the representative example, where *α*_*w*_ = 0.05, are illustrated in Figure 2(f)–(h). In notations of the electric/magnetic multipolar coefficients, *a*_*e*/*m*_(*l*, *m*), *l* stands for the multipolar order, and *m* is the magnetic number, which characterizes the orientation of the orbitals. According to the spectrum of the multipolar contributions in the vicinity of the resonant wavelength 920 nm, the dominant multipolar coefficients are *a*_*e*_(1, 0), *a*_*m*_(1, ±1), and *a*_*m*_(2, ±2). The extracted multipolar amplitudes for the quasi-BIC eigenmode at the wavelength of 920 nm are visualized by histograms in Figure 2(g) and (h). The multipoles *a*_*e*_(1, 0) and *a*_*m*_(2, ±2) with zero and even *m* do not lead to the out-of-plane radiation, constituting the nature of the symmetry-protected BIC state. This result is because the *z*-propagating plane waves contain multipoles exclusively with *m* = ±1.

The asymmetry-induced in-plane magnetic dipole moment *m*_*y*_, responsible for the radiative losses and limiting the Q factor, stems from the nonzero combination of the coefficients *a*_*m*_(1, ±1), $m_{y}\propto \left[a_{m}\left(1,-1\right)+a_{m}\left(1,1\right)\right]$ . Our calculations show that the terms *a*_*m*_(1, ±1) exhibit rapid growth as the asymmetry parameter increases, rapidly destroying the perfect BIC state. Detailed simulation of electric and magnetic multipoles for different geometric asymmetries from 0 (non-radiative BIC case) to 10 % are shown in section S2 of the Supplementary.

Having independently calculated the coupling coefficients for both Δ*w* and Δ*ɛ* cases, the right-hand side of Eq. (1) yields a nearly constant value of about 0.4. This finding demonstrates the more substantial influence of the structural disbalance in the quasi-BIC radiative loss and is illustrated in Figure 2(e) with the orange dots. It matches well with the blue line plotted based on the Q factors retrieved from the complex-valued eigenfrequencies in the numerical modelling (see the complete data in section S3 of the Supplementary). In general, this ratio consistently found from Eqs. (1) and (2) can be regarded as a practical guideline for dynamic compensation strategies in metasurfaces incorporating both the geometric asymmetry and optically-induced permittivity variations.

### 2.3 Simulations of bandwidth modulation

**Figure 3:**
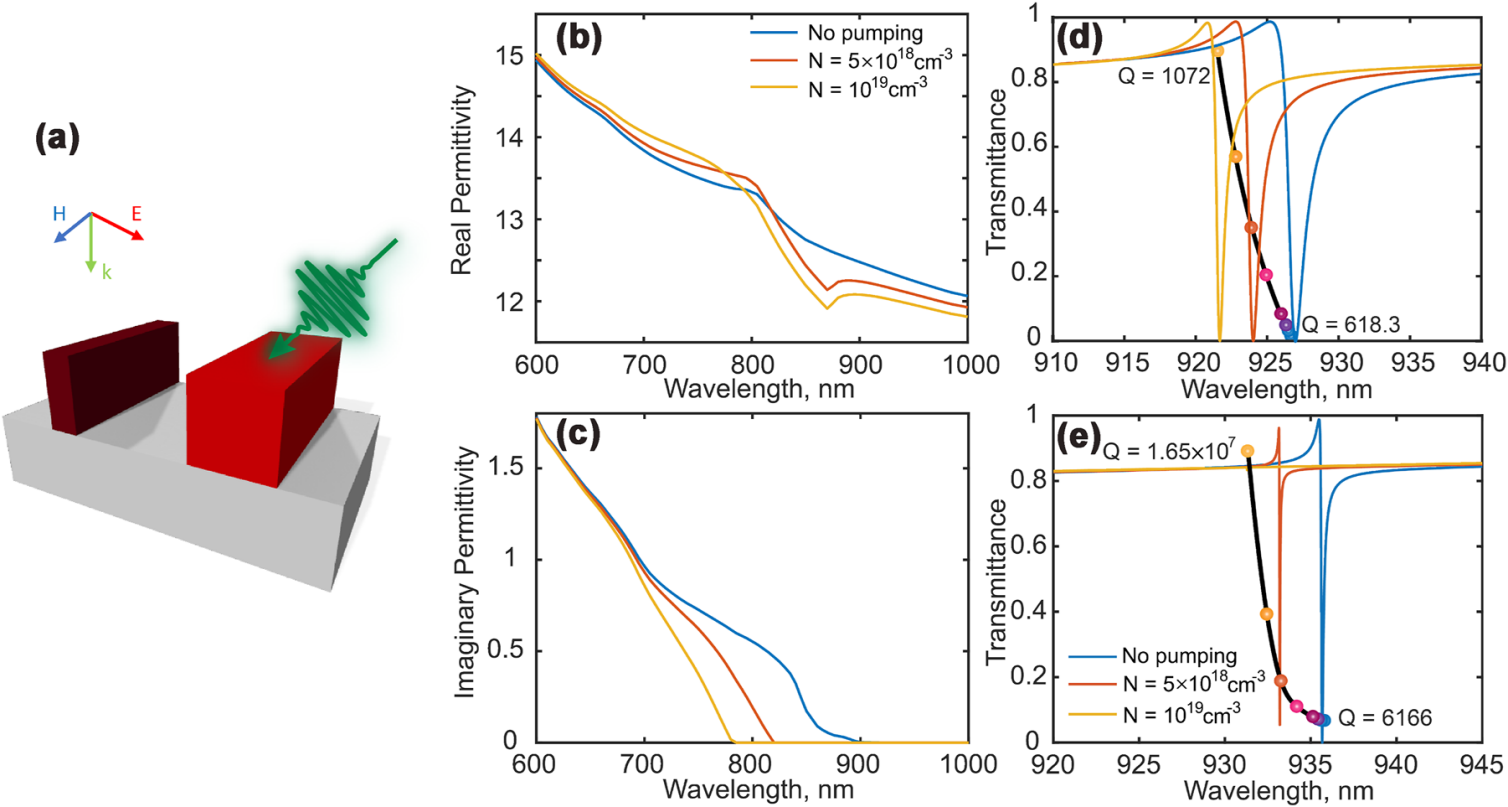
Material (GaAs) and metasurface optical properties at different carrier densities. (a) Schematic of the unit cell with a free carrier induced permittivity disbalance under the right-side nano-bar pumping. (b) and (c) Show the real and imaginary parts of the permittivity spectrum for different carrier densities. The blue curve indicates the static state when the metasurface is not pumped. The orange and yellow curves in (b) and (c) demonstrate the permittivity under 5 × 10^18 ^cm^−3^ and 1 × 10^19 ^cm^−3^ carrier density injection, correspondingly. (d) and (e) Show the transmittance spectrum with different free carrier density injections under the right side grating pumping. (d) Is the resonance transmittance spectrum designed for *α*_*w*_ = 5 %. The Q-factor increases from 618.3 to 1072 under 1 × 10^19 ^cm^−3^ free carrier concentration. The spectrum (e) is calculated for *α*_*w*_ = 1.6 %, and shows this quasi-BIC-type resonance (*Q* = 6, 166) transitioning to a pure BIC state (*Q* = 1.65 × 10^7^) under the pumping.

In this section, we perform full simulations with an experimentally realistic set of material parameters and models that have been verified in prior research [25], [33], [34]. For consistency, we maintain the same structure parameters, including metagrating height, width, and unit cell periodicity, as shown in Figure 3(a). We have also included a transparent SiO_2_ substrate with a refractive index of 1.45. The inclusion of the substrate results in a decrease in the metasurface Q-factor for small disbalance ratio (*α*_*w*_ < 0.005, see Figure S3(a) of the Supplementary), as expected [35]. For larger disbalance ratios (larger *α*_*w*_), the Q-factor of the metasurface is not significantly altered.

As mentioned above, the free carriers will occupy the lower energy levels of the conduction band when the optical pumping energy exceeds the bandgap of GaAs. This phenomenon results in a modification of permittivity proportional to the free carrier concentration. To accurately describe the change in the real and imaginary part of the GaAs permittivity, we cannot solely rely on the Drude model description. We, therefore, incorporate an additional effect that occurs in proximity to the material’s bandgap under optical pumping conditions, known as the band-filling effect. When the free carriers occupy the lower-level energy states of the conduction band, the transition from the valance band is not allowed. This will decrease the absorption of light beams with energy near the bandgap. Note that the GaAs material can still exhibit a significant absorption loss when the probe light has energy larger than its bandgap. As a result of this absorption, the Q-factor of the metasurface modes with energy above the bandgap will be significantly reduced. Thus, our probe pulses should have energy not far from the bandgap but close enough to be impacted by the band-filling effect. Specifically, we chose probe pulses in the wavelength range of 900 − 1, 000 nm.

Based on both the Drude model and band-filling contribution (in the region *λ* > 900 nm), the permittivity change is

(3) $$\Delta \varepsilon =\Delta \varepsilon_{\text{BF}}+\Delta \varepsilon_{\text{D}}<0,$$

where Δ*ɛ*_BF_ is the contribution from the band-filling effect and Δ*ɛ*_D_ is from the Drude model. Both of them give a negative permittivity change. We note that the band-filling contribution dominates other contributions, such as the one from the bandgap shrinkage effect [33], which are correspondingly neglected in our model. A detailed explanation of both contributions is given in section S4 of the Supplementary.

We next compute the permittivity change for different free carrier densities, as illustrated in Figure 3(b) and (c). In Figure 3(b), we present the real part of the permittivity for three different carrier densities. No pumping indicates the original static state of the GaAs, where the value of the GaAs permittivity is experimentally measured. For carrier densities of *N* = 5 × 10^18^ cm^−3^ and *N* = 1 × 10^19^ cm^−3^, the permittivity is significantly modified in the spectral region near the GaAs bandgap. Figure 3(c) shows the imaginary part of the GaAs. It reveals how increasing the carrier density significantly reduces the material’s absorption, resulting in negligible non-radiative losses in the spectral region 900 − 1, 000 nm compared to shorter wavelengths.

Next, we vary the carrier density for the right-side nano-bar from *N* = 0 (no pumping) to *N* = 1 × 10^19^ cm^−3^ while keeping the permittivity of the left-side nano-bar unchanged. Figure 3(d) and (e) presents the simulated transmittance spectra. Figure 3(d) corresponds to the case when the left-side nano-bar is 5 % narrower than the right-side nano-bar. The quasi-BIC resonance exhibits a blue shift as the carrier density increases. Meanwhile, as indicated in the figure, the Q-factor of the resonance will increase from 618.3 to 1072. That is attributed to the left-side nano-bar having a smaller width. When the pumping occurs, the permittivity of the right-side nano-bar will decrease, as described in Eq. (4), compensating for the left-side grating and reducing the disbalance while moving closer to the BIC condition.

If the initial static asymmetry is low, the quasi-BIC mode can transform to a pure BIC mode, e.g., the dynamic asymmetry can fully balance the static one. Figure 3(e) depicts the simulated transmittance spectrum for this case. Leveraging the predicted Q-factor ratio, calculated in Section 2.2, we determine that the induced Δ*ɛ* ≈ 0.32 at a carrier density *N* = 1 × 10^19^ cm^−3^, accounting for 2.5 % change of the original permittivity. Furthermore, the amplitude of the magnetic dipole provides a linear relation for the small perturbation, Δ*ɛ* and Δ*w*. Calculated in Eq. (1) and S2 of the Supplementary, the ratio of the magnetic dipole amplitude between Δ*ɛ* and Δ*w* is approximately 3/2. To fully compensate for the structural asymmetry caused by the different widths of the nano-bars, the ratio of the two Q-factor disbalance terms should equal one. We can then find the approximate ratio *α*_*w*_ ≈ 0.016. Applying this number to our simulations shows that the transmittance spectrum becomes fully flat at high carrier density. This flat spectrum indicates that the Q-factor of this mode goes to infinity, corresponding to pure BIC mode. These findings demonstrate that we can dynamically close the resonance using a structured pump illumination when we selectively excite the right-side nano-bar. In contrast, if we excite the left-side nano-bar, we will increase the disbalance and broaden the resonance bandwidth, as shown in section S5 of the Supplementary.

### 2.4 Time-varying metasurface in the Q-boosting regime

Next, we study the dynamic manipulation of probe pulses in the Q-boosting regime to demonstrate efficient frequency conversion and light trapping. The dynamic modulation of the permittivity of the GaAs is driven by free-carrier excitation, which happens on tens of femtosecond timescale. First, we present a mathematical model that describes the dynamic modulation of the resonance state. Subsequently, we utilize finite-difference time-domain (FDTD) simulations and a temporal coupled mode theory (TCMT) to reveal and mutually verify the bandwidth modulation and light trapping phenomenon.

To describe the dynamic switch between two different carrier density states, we use a simplified model based on a two-level rate equation

(4) $$\frac{\text{d}}{\text{d}t}N\left(t\right)=A\exp\left(-\frac{t^{2}}{\tau_{c}^{2}}\right)-rN\left(t\right),$$

where *τ*_*c*_ is the rising speed of the free-carrier excitation, and *r* = 0.1 is their recombination rate. *A* is a constant proportional to the pump intensity and absorption.

**Figure 4:**
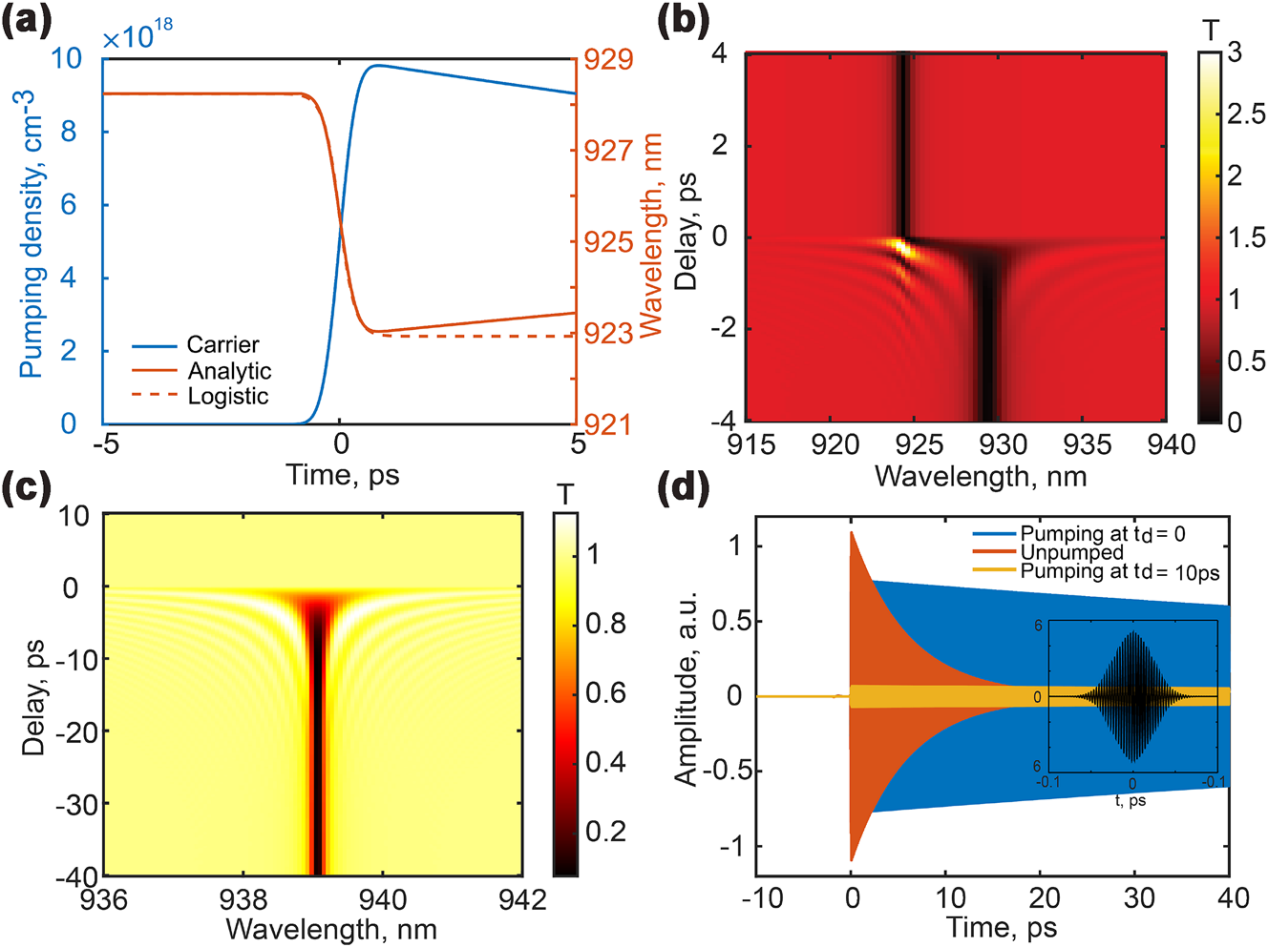
Ultrafast metasurface response. The blue line of (a) shows the free-carrier density obtained using a two-level system with a Gaussian pulse in the time domain. The red solid line shows the calculated resonance shift. The line shape of this solution can be expressed by a logistic function, shown in a red dashed line. (b) Demonstrates time-dependent transmittance from unpumped to pump state, having carrier density up to 10^19^ cases. The resonance exhibits Q-boosting during the pumping due to the permittivity and width compensation. (c) Demonstrates the light trapping for the full compensation between the static and dynamic asymmetry. Its amplitude damping in time scale is shown in (d); the inset with a black line is the incident Gaussian pulse with arbitrary amplitude.

Because GaAs has a much longer recombination time (low recombination rate) than the free-carrier excitation time, we can assume that the free-carrier concentration remains nearly constant after the pump pulse. In Figure 4(a), the blue curve presents the carrier density profile versus time. The time zero on the *x*-axis corresponds to the center of the Gaussian pulse used to model the pumping energy distribution. By substituting the carrier density term in the permittivity model, see details in section S6 of the Supplementary, we can obtain the time dependence of the permittivity as a logistic-type function.

Next, we evaluate the quasi-BIC resonance shift due to this permittivity change. This is obtained using the first-order perturbation theory (see details in section S6 of the Supplementary). As such, the resulting perturbed equation (perturbed eigenvalues) can be solved order by order utilizing only the unperturbed operator. Hence, in our case, using the Δ*ɛ* as the perturbation strength, the wavelength shift can be expressed as an integration of the unperturbed mode electric field multiplied by Δ*ɛ*, involving an error function [36] (see Eq. (S18) of the Supplementary). We can, then, obtain the function for the resonance position with time, shown by the red solid line in Figure 4(a). The wavelength shifts from 928 nm to 923 nm for less than one picosecond.

Notably, to visualize the recombination effect in the model, we have plotted the curve using a recombination time of 50 ps, which is much shorter than the realistic lifetime of electrons in GaAs, typically of the order of 5 ns [37]. In practice, the free-carrier density should exhibit a flatter recombination dependence than the red solid line in Figure 4(a).

After obtaining the dependence of the resonant wavelength [*λ*(*t*)] and Q-factor [*Q*(*t*)] with time, we can develop a temporal coupled mode theory (TCMT)

(5) $$\dot{a}\left(t\right)=-\left[i\omega \left(t\right)+\gamma \left(t\right)\right]a\left(t\right)+\sqrt{\gamma \left(t\right)}s^{+}\left(t\right).$$

Here, the *a*(*t*) is the complex amplitude of the designed mode, and *ω* = 2*πc*/*λ* and *γ* = *c*/*Qλ* are the angular frequency and loss rate (radiative plus non-radiative), respectively, as functions of time. *c* stands for the speed of light. The external excitation, probe pulse, in this ordinary differential equation, shown as *s* ^+^(*t*), is modeled as a Gaussian pulse $s^{+}\left(t\right)=A\left(t\right)e^{-i\omega_{\text{i}}t}$ , where the amplitude is $A\left(t\right)=A_{0}\exp\left(-\frac{t^{2}}{\tau_{\text{i}}^{2}}\right)$ and *τ*_i_ is the pulse duration.

In our case, we use a broadband probe pulse centered at 920 nm, with a pulse duration *τ*_*i*_ = 20*π*/*ω*_*i*_, where *ω*_*i*_ represents the angular frequency. The probe pulse is centered at *t* = 0. Next, we use an eigenmode solver, COMSOL, to extract the mode information for the eigenfrequency and damping coefficient (related to the resonant wavelength and Q-factor). The initial wavelength of the mode is found to be *λ* = 928 nm with a Q-factor *Q* = 618.3. As a result of the pumping process, the wavelength shifts to 923 nm, and its Q-factor is boosted to *Q* = 1, 072. We model the density of free carriers induced by the pump pulse as a logistic function, shown in S6.2 of the Supplementary. The delay time between the probe and pump pulses is Δ*t* = *t*_Probe_ − *t*_Pump_.

With these parameters, we compute the transmittance spectrum of the metagrating at different time delays, shown in Figure 4(b). When the pump arrives after the probe pulse, the spectrum remains unchanged because there is no interaction between the pump and probe, from −∞ to −2 ps. When the pump arrives before the probe, the probe will detect the excited mode, increasing the decay time of the mode and, correspondingly, its Q-factor, for delays from 1 ps to ∞. However, as the pump approaches the probe, part of the input probe pulse energy will couple to the quasi-BIC mode. At the same time, the mode will shift in frequency, and the coupled energy will be captured in this high-Q mode. This Q-boosting effect at delays −2 − 0 ps will transfer a large portion of the input probe energy into new frequencies, thereby generating an effective gain for these frequencies. This effective gain can be seen in Figure 4(b) as transmissivity that is larger than unity, *T* ≈ 2.5, at delay Δ*t* ≈ 0.

After performing calculations based on TCMT, we verified them using the Lumerical FDTD simulations. The FDTD Lumerical provides a Flexible Material Plugins function, which modifies the time domain update equations to change the permittivity as a function of time. Here, we modelled the material permittivity as a step-function in time. For example, the probe pulse launch time was set at 4 ps, and the step-function, regarded as the permittivity change by the free carrier injection, switched spot varied from 0 to 8 ps to create the time delay between the pump and probe. The comparison of results between the TCMT and the FDTD method is presented in section S7 of the Supplementary, demonstrating a solid agreement.

When calculating the Q-boosting effect, we also explored the possibility of achieving perfect dynamic compensation between the Δ*ɛ* and Δ*w*. This compensation would close the resonance and eliminate the radiative loss to obtain an infinity Q-factor. The initial resonant wavelength of the quasi-BIC mode is 939 nm, and its lifetime is at 50*π*/*ω*. After the pumping, the BIC mode shifts to a wavelength of 938 nm and has *Q* = 10^7^. Using our TCMT, we obtained the transmittance spectrum in both frequency and time domain, shown in Figure 4(c) and (d). In this case, the increased transmittance happens simultaneously on both sides of the resonance. The larger-than-unity transmittance is only observed before time zero. After the zero delay, the resonance is entirely closed and does not couple to the free-space probe pulse.

Furthermore, we show the time dynamics of the probe pulse for three time-delays, corresponding to no-pumping, zero delay, and 10 ps time delay. The orange curve in Figure 4(d) indicates the dynamics of the initial state with a significant decay rate, labeled as the mode under unpumped conditions. The yellow curve shows the final BIC state, labeled as pump delay at 10 ps. In this situation, the damping of the mode lasts tens of picoseconds. However, this ’dark state’ cannot be excited and coupled into the free space. Thus, this state shows a low amplitude. When the pump pulse is close to the probe pulse, the blue curve, the mode is excited efficiently with a very high amplitude, accompanied by a long resonance lifetime. The inset of Figure 4(d) demonstrates an incident probe pulse.

## 3 Conclusion and discussion

In conclusion, our work proposes and numerically demonstrates a novel way of Q-boosting in semiconductor metasurface driven by a quasi-BIC symmetry-protected mode. We demonstrate a significant Q-boosting effect at the femtosecond scale by dynamic compensation of the static in-plane geometric asymmetry through all-optical permittivity modulation by a structured pump. Unlike the dual-material metasurfaces Q-boosting mechanism, our quasi-BIC mode’s design is only determined by the geometric asymmetry, utilizing the band-filling effect to eliminate absorption losses and achieve complete conversion between quasi-BIC to BIC mode. To explain this phenomenon, we developed an analytical model based on a multipolar decomposition to demonstrate the mode response influenced by geometric asymmetry and permittivity disbalance. We find that the contribution of these two factors to the mode depends on its type. In our case, a 1.6 % geometric asymmetry ratio requires a 2.5 % permittivity disbalance ratio to compensate. Thus, we can use the calculated maximum dielectric constant change to infer the bar width of the metasurface that should be designed to achieve complete mode conversion.

Furthermore, we developed a dynamic model based on TCMT to trace the time dynamics of the GaAs metasurface. We show how the coupling between broadband light and resonator has a remarkable enhancement at the time-varying regime. Also, we found this mode enhancement can trap the light over several picoseconds under a complete BIC condition. In the frequency domain, a transmittance enhancement (>1) appears at the transmittance spectrum, and this value is dependent on the material response of free carrier accumulation.

Our results demonstrate a novel way to engineer the dynamic time-bandwidth response of time-variant metasurfaces by balancing their geometric and dynamic asymmetries. These findings can find important potential practical applications in nonlinear frequency conversion and light trapping.

## Supplementary Material

Supplementary Material Details
