# Supplementary material for: Ultrafast Q-boosting in semiconductor metasurfaces
Source: Nanophotonics. 2024 Feb 19;13(12):2173–82. doi: 10.1515/nanoph-2023-0718 (PMC11501241; doi:10.1515/nanoph-2023-0718)
Supplement: Supplementary file 1 — Supplementary Material Details [file j_nanoph-2023-0718_suppl_001.pdf]

Supplementary

Ziwei Yang*, Mingkai Liu, Daria Smirnova, Andrei Komar, Maxim Shcherbakov, Thomas Pertsch, and Dragomir Neshev*

Ultrafast Q-boosting in semiconductor metasurfaces

S1 Multipolar representation of the far-field coupling coefficient

In the coupled-mode-theory, the inverse radiation lifetime γ_{rad} of the mode is related to the far-field coupling coefficients $D_{x,y}$ in the x and y polarization radiation channels as follows (see, e.g., Refs. [1, 2]),

$$\gamma_{\text{rad}}/c = |D_x|^2 + |D_y|^2 . \tag{S1}$$

We assume the material is lossless and the quality factor of the mode is determined solely by the radiative losses,

$$Q = \frac{\omega_0}{\gamma_{\text{rad}}} = \frac{\omega_0}{|D_{x,y}|^2 c}, \tag{S2}$$

where ω_0 is the angular frequency of the resonant state and c is the speed of light. Therefore, we can relate the quality factors of the quasi-BIC modes with finite lifetimes due to the geometric or permittivity asymmetry to each other by calculating the ratio,

$$\frac{Q(\Delta w)}{Q(\Delta\varepsilon)} = \left[\frac{D_{x,y}(\Delta\varepsilon)}{D_{x,y}(\Delta w)}\right]^2 . \tag{S3}$$

In turn, the coupling coefficient can be represented as a superposition of the multipolar contributions to the radiation out-of-plane, with the leading terms being the components of the $\boldsymbol{p}$ (electric dipole), $\boldsymbol{m}$ (magnetic dipole), $\boldsymbol{Q^e}$ (electric quadrupole) and $\boldsymbol{Q^m}$ (magnetic quadrupole):

$$D_x = -\frac{k_0}{\sqrt{2S_0}}\left[p_x - \frac{m_y}{c} + \frac{ik_0}{6}Q^e_{xz} + \frac{ik_0}{6c}Q^m_{xz} + \cdots\right] , \tag{S5a}$$

$$D_y = -\frac{k_0}{\sqrt{2S_0}}\left[p_y + \frac{m_x}{c} + \frac{ik_0}{6}Q^e_{yz} + \frac{ik_0}{6c}Q^m_{yz} + \cdots\right] . \tag{S5b}$$

***Corresponding author: Ziwei Yang,** ARC Centre of Excellence for Transformative Meta-Optical Systems (TMOS), Dept. of Electronics Materials Engineering, Research School of Physics, Australian National University, ACT 2600, Australia, e-mail: Ziwei.Yang@anu.edu.au

***Corresponding author: Ziwei Yang,** Institute of Applied Physics, Abbe Center of Photonics, Friedrich Schiller University Jena, Albert-Einstein-Straße 15, 07745 Jena, Germany

Mingkai Liu, Daria Smirnova, Andrei Komar, ARC Centre of Excellence for Transformative Meta-Optical Systems (TMOS), Dept. of Electronics Materials Engineering, Research School of Physics, Australian National University, ACT 2600, Australia

Maxim Shcherbakov, Department of Electrical Engineering and Computer Science, University of California, Irvine, CA 92697, USA

Thomas Pertsch, Institute of Applied Physics, Abbe Center of Photonics, Friedrich Schiller University Jena, Albert-Einstein-Straße 15, 07745 Jena, Germany

***Corresponding author: Dragomir Neshev,** ARC Centre of Excellence for Transformative Meta-Optical Systems (TMOS), Dept. of Electronics Materials Engineering, Research School of Physics, Australian National University, ACT 2600, Australia, e-mail: Dragomir.Neshev@anu.edu.au

The multipolar moments can be expressed through the spherical multipole coefficients [3, 4]. For the electric dipole moment,

$$\begin{aligned} p_x^\omega &= C_1^e \frac{a_e^\omega(1,-1) - a_e^\omega(1,1)}{\sqrt{2}}, \\ p_y^\omega &= C_1^e \frac{a_e^\omega(1,-1) + a_e^\omega(1,1)}{\sqrt{2}i}. \end{aligned} \tag{S6}$$

For magnetic dipole moment,

$$\begin{aligned} m_x^\omega &= C_1^m \frac{a_m^\omega(1,-1) - a_m^\omega(1,1)}{\sqrt{2}}, \\ m_y^\omega &= C_1^m \frac{a_m^\omega(1,-1) + a_m^\omega(1,1)}{\sqrt{2}i}. \end{aligned} \tag{S7}$$

For magnetic quadrupole,

$$\begin{aligned} Q_{xz}^m = Q_{zx}^m &= C_2^m \left[\frac{a_m^\omega(2,-1) - a_m^\omega(2,1)}{2}\right], \\ Q_{yz}^m = Q_{zy}^m &= C_2^m \left[\frac{a_m^\omega(2,-1) + a_m^\omega(2,1)}{2i}\right]. \end{aligned} \tag{S8}$$

For the electric quadrupole,

$$\begin{aligned} Q_{xz}^e = Q_{zx}^e &\approx C_2^e \left[\frac{a_e^\omega(2,-1) - a_e^\omega(2,1)}{2}\right], \\ Q_{yz}^e = Q_{zy}^e &\approx C_2^e \left[\frac{a_e^\omega(2,-1) + a_e^\omega(2,1)}{2i}\right]. \end{aligned} \tag{S9}$$

The coefficients are $C_1^e = \frac{\sqrt{3}\pi}{i\omega_0}$, $C_1^m = -\frac{\sqrt{3}\pi}{k_0}$, $C_2^\mathrm{e} = \frac{6\pi\sqrt{10}}{ck_0^2}$ and $C_2^\mathrm{m} = \frac{6\pi\sqrt{10}}{ik_0^2}$. In this way, we can determine all the multipolar radiation channels for the quasi-BIC mode.

S2 Multipolar moments for different in-plane symmetry breaking rates

To figure out the dominant radiation channel of the quasi-BIC mode, we plot all the calculated multipolar contributions in Figs. S1(a) and leading multipolar moments in Fig. S1(b). We observe the in-plane magnetic dipole moment rapidly increases with increasing asymmetry. Its growth rate coincides with a rapid decrease in the quality factor of the mode. Figure S1(c) shows the spectrum of multipolar contributions for $\alpha_w = 1\%$, wherein the multipoles are spectrally narrower, but the set of leading multipoles remains the same.

S3 Data for Q-factor ratio and band calculation

In Fig. 2 of the main text, we plot the quality factors of the mode vs the asymmetry parameter introduced by two different means, α_ϵ and α_w. In our calculations, we used the eigenmode solver of COMSOL Multiphysics, which is implemented in the 3D finite-element method. The model setting includes one unit cell of the metasurface, with Floquet-periodic boundary conditions (assuming the Bloch wave number k_x) imposed in the x-direction and continuity boundary conditions in the y-direction. Perfectly matched layers were used in the out-of-plane ($\pm z$) direction (the domain size is specified as twice the characteristic wavelength). After the meshing process, the complex-valued eigenfrequency is obtained. From the imaginary part of the eigenmode frequency, we correspondingly calculate the loss rate and the Q-factor. The coupling amplitudes squared $D(\Delta w), D(\Delta\varepsilon)$ are reconstructed from the

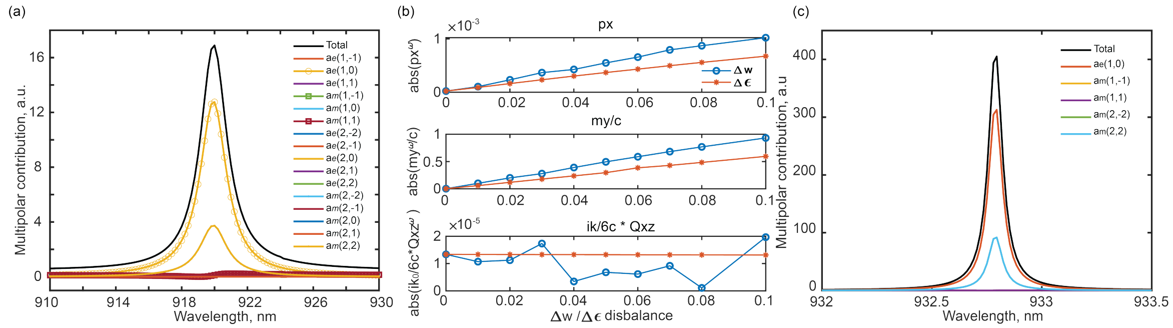

Fig. S1: (a) Spectrum of the multipolar contributions near the quasi-BIC resonance for $\alpha_w = 5\%$, and (b) multipolar moments depending on the asymmetry parameter. (c) Spectrum of the multipolar contributions for $\alpha_w = 1\%$ ($Q = 12,299$).

multipolar decomposition of the electromagnetic field. The detailed data are presented in Tables 1, 2 below.

Tab. 1: Q-factor of the quasi-BIC mode for different α_w and α_ϵ.

Terms	0	0.01	0.02	0.03	0.04	0.05	0.06	0.07	0.08	0.1
$Q(\Delta w)$	5.068e12	12299	3041.6	1337.9	745.18	472.02	324.63	236.27	179.14	112.56
$Q(\Delta\epsilon)$	5.068e12	31464	7842.7	3473.4	1946.6	1241.2	858.77	628.58	479.47	304.62

Tab. 2: Coupling amplitude squared for different α_w and α_ϵ.

Terms	0	0.01	0.02	0.03	0.04	0.05	0.06	0.07	0.08	0.1
$D(\Delta w)$	2.599e-2	1.0054	2.0058	2.7842	3.905	4.9483	5.8806	6.8164	7.6863	9.3278
$D(\Delta\epsilon)$	2.599e-2	0.5869	1.1881	1.7849	2.3711	2.9655	3.8494	4.2971	4.8414	5.9472

After that, we calculated the band structure in the vicinity of the designed mode, as shown in Figure S2(a). The red curve corresponds to the mode utilized in our work. The BIC point occurs at $k_x = 0$, which is the Γ point of the Brillouin zone. We also track the Q factor divergence of the utilized mode, shown in S2(b). The Q-factor of the mode decreases from 10^{13} to 10^8 with the k_x far away from the Γ point.

We also investigated the influence of the substrate on the Q-factor of the metagrating as a function of its geometric disbalance. As shown by the orange curve in Figure S3, the Q-factor of the structure on a SiO_2 substrate is not significantly affected. The blue curve corresponds to the Q-factor of the metagrating without a substrate, as also given in the Table 1 of S3. We note that the substrate (see the figure on the right for a metagrating on a substrate with $n_{\text{sub}} = 1.45$) will preserve the contributions of the different multipoles, similar to the case without a substrate.

S4 Refractive index model

In this section, we will introduce the model of complex refractive indices calculation from the change in carrier density for direct bandgap semiconductors like GaAs, InP, and InGaAsP [5–7]. We start

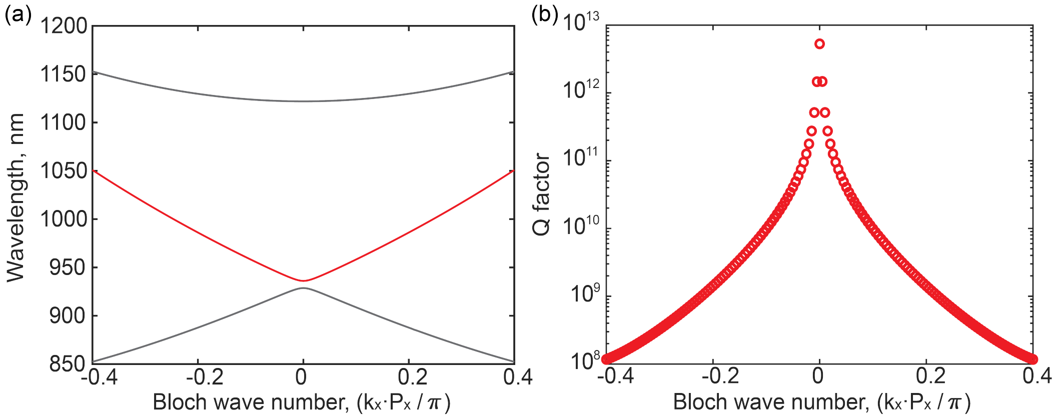

Fig. S2: (a) Band structure of the designed metagrating. The red curve denotes the designed magnetic dipole-dominated mode. (b) Q-factor of designed mode.

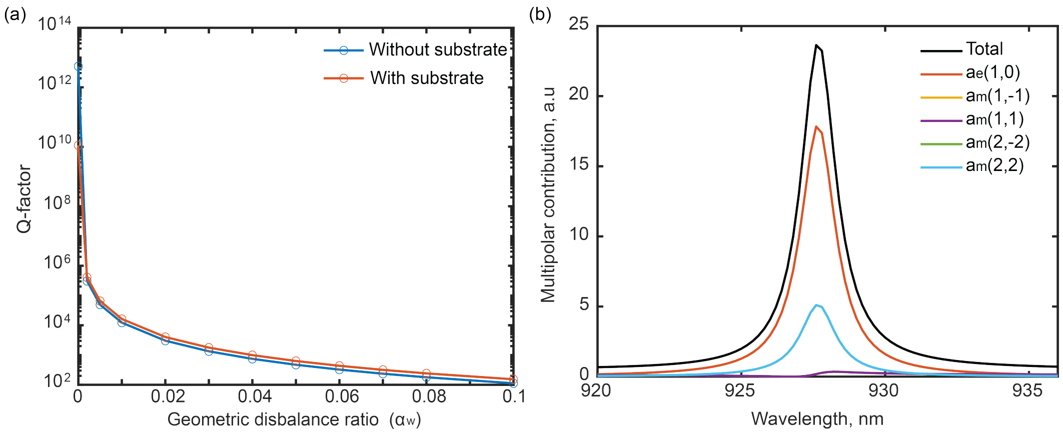

Fig. S3: (a) Q-factor of two metagratings with different disbalance rates. (b) The spectrum of the multipolar contributions near the quasi-BIC resonance for $\alpha_w = 5\%$ with a substrate of $n_{\mathrm{sub}} = 1.45$.

with a general equation described in the main text but with a refractive index scale,

$$\Delta n = \Delta n_{\mathrm{BF}} + \Delta n_{\mathrm{D}} + \Delta n_{\mathrm{BS}} < 0 \tag{S10}$$

From the equation, we notice that the change in the refractive index is dependent on three factors: Drude term, Band Filling term and band shrinkage term. However, the magnitude of the band shrinkage term is several orders lower than the rest of the two terms. To simplify the equation in the calculation, we will not discuss the band shrinkage term in this paper.

For the well-known model in the semiconductor carrier modeling, we give the refractive index change and loss change of the Drude model in the following equations [5, 8];

$$\begin{aligned} \Delta n_D &= -\left(\frac{e^2\lambda^2}{8\pi^2 c^2 \varepsilon_0 n_0}\right)\left[\frac{N_e}{m_e} + N_h\left(\frac{m_{hh}^{0.5}+m_{lh}^{0.5}}{m_{hh}^{1.5}+m_{lh}^{1.5}}\right)\right], \\ \Delta \alpha_D &= \left(\frac{e^3\lambda^2}{4\pi^2 c^3 \varepsilon_0 n_0}\right)\left[\frac{N_e}{m_e^2\mu_e} + \frac{N_h}{\mu_h}\left(\frac{m_{hh}^{0.5}+m_{lh}^{0.5}}{m_{hh}^{1.5}+m_{lh}^{1.5}}\right)^2\right], \end{aligned} \tag{S11}$$

where N_e and N_h stand for changing the densities of the electrons and holes; m_e, m_{lh} and m_{hh} are the masses of electrons, light holes, and heavy holes; μ_e and μ_h are described as the electrons and holes mobility. Also, e is the elementary charge, ϵ_0 is vacuum permittivity, and n_0 is the unperturbed refractive index.

Then, we also model the band-filling effect, which is only dominant near the bandgap. The interband transitions define such an effect, and the equations of refractive index and absorption change are demonstrated as follows;

$$\Delta n_{\mathrm{BF}}(N,E,T)=\frac{2c\hbar}{e^2}\mathrm{PV}\int_0^\infty\frac{\Delta\alpha_{BF}\left(N,E',T\right)}{E'^2-E^2}\,\mathrm{d}E', \tag{S12}$$

where the absorption term in the S12 $\Delta\alpha_{BF}$ is

$$\Delta\alpha_{BF}=\frac{C_{\mathrm{hh}}}{E}\sqrt{E-E_{\mathrm{g}}}\left[f_{\mathrm{v}}\left(E_{\mathrm{ah}}\right)-f_{\mathrm{c}}\left(E_{\mathrm{bh}}\right)-1\right]+\frac{C_{\mathrm{lh}}}{E}\sqrt{E-E_{\mathrm{g}}}\left[f_{\mathrm{v}}\left(E_{\mathrm{al}}\right)-f_{\mathrm{c}}\left(E_{\mathrm{bl}}\right)-1\right], \tag{S13}$$

For Equation S12, E is denoted as photon energy, and PV indicates the Cauchy principal value of the integral. And Equation S13 describes the interband absorption, which only exists when the photon energy is smaller than the bandgap energy.

For the material we used in the paper, GaAs, $C_{hh}=3.1\times10^6cm^{-1}eV^{\frac{1}{2}}$ and $C_{lh}=1.6\times10^6cm^{-1}eV^{\frac{1}{2}}$. Additional, $f_{\mathrm{v}}\left(E_{\mathrm{ah,al}}\right)$ and $f_{\mathrm{c}}\left(E_{\mathrm{bh,bl}}\right)$ are the Fermi-Dirac distributions and can be described by the following Equation S14;

$$\begin{aligned}f_{\mathrm{c}}\left(E_{\mathrm{bh,bl}}\right)&=\frac{1}{1+\exp\left(\frac{E_{\mathrm{bh,bl}}-E_{\mathrm{Fc}}}{k_{\mathrm{B}}T}\right)},\\ f_{\mathrm{v}}\left(E_{\mathrm{ah,al}}\right)&=\frac{1}{1+\exp\left(\frac{E_{\mathrm{ah,al}}-E_{\mathrm{Fv}}}{k_{\mathrm{B}}T}\right)},\end{aligned} \tag{S14}$$

here, the approximation of quasi-Fermi levels $E_{\mathrm{F_c}}$ and $E_{\mathrm{F_v}}$ are described by the densities state of conduction and valence band, $N_{\mathrm{c}}=2\left(\frac{m_{\mathrm{e}}k_{\mathrm{B}}T}{2\pi\hbar^2}\right)^{3/2}$ and $N_{\mathrm{c}}=2\left(\frac{m_{\mathrm{dh}}k_{\mathrm{B}}T}{2\pi\hbar^2}\right)^{3/2}$.

$$\begin{aligned}E_{\mathrm{F_c}}&=\left[\ln\left(\frac{N_{\mathrm{e}}}{N_{\mathrm{c}}}\right)+\frac{N_{\mathrm{e}}}{N_{\mathrm{c}}}\left[64+0.05524\frac{N_{\mathrm{e}}}{N_{\mathrm{c}}}\left(64+\sqrt{\frac{N_{\mathrm{e}}}{N_{\mathrm{c}}}}\right)\right]^{-1/4}\right]k_{\mathrm{B}}T,\\ E_{\mathrm{F_v}}&=-\left[\ln\left(\frac{N_{\mathrm{p}}}{N_{\mathrm{v}}}\right)+\frac{N_{\mathrm{p}}}{N_{\mathrm{v}}}\left[64+0.05524\frac{N_{\mathrm{p}}}{N_{\mathrm{v}}}\left(64+\sqrt{\frac{N_{\mathrm{p}}}{N_{\mathrm{v}}}}\right)\right]^{-1/4}\right]k_{\mathrm{B}}T-E_{\mathrm{g}},\end{aligned} \tag{S15}$$

where the density-of-state effective mass for holes is $m_{dh}=(m_{hh}^{\frac{3}{2}}+m_{lh}^{\frac{3}{2}})^{\frac{2}{3}}$.

For the ideal assumption, there will be two values for each band because of the degeneracy of the conduction and valence bands. Then we get:

$$\begin{aligned}E_{\mathrm{ah,al}}&=\left(E_{\mathrm{g}}-E\right)\left(\frac{m_{\mathrm{e}}}{m_{\mathrm{e}}+m_{\mathrm{hh,lh}}}\right)-E_{\mathrm{g}},\\ E_{\mathrm{bh,bl}}&=\left(E-E_{\mathrm{g}}\right)\left(\frac{m_{\mathrm{hh,lh}}}{m_{\mathrm{e}}+m_{\mathrm{hh,lh}}}\right).\end{aligned} \tag{S16}$$

By substituting the S14, S15 and S16, we will obtain the $\Delta n(\lambda)$ and absorption extinction coefficient change $\Delta k(\lambda)=\frac{\lambda\Delta\alpha}{4\pi}$.

S5 Metasurface design and left-side nano-bar pumping

Instead of using the ideal geometric model (without the substrate), we design the metasurface in the main text 2.3 with a SiO_2 substrate, $n=1.46$. With the consistency of the ideal model, we fixed the parameters of the structure as main text 2.2 described. We first did a parameter sweeping of the geometric disbalance rate from -10% to 10%, shown in Figure S4(b). This in-plane symmetry BIC

mode is leaked out by squeezing the left bar with a certain percentage decrease compared with the right bar artificially, and the mode Q-factor is proportional to the -2 power of α_w. After choosing the designed bandwidth, we also investigated the influence of the width of the nano-bar. We found the width only shifts the resonance wavelength but with a little on the Q-factor, Figure S4(c).

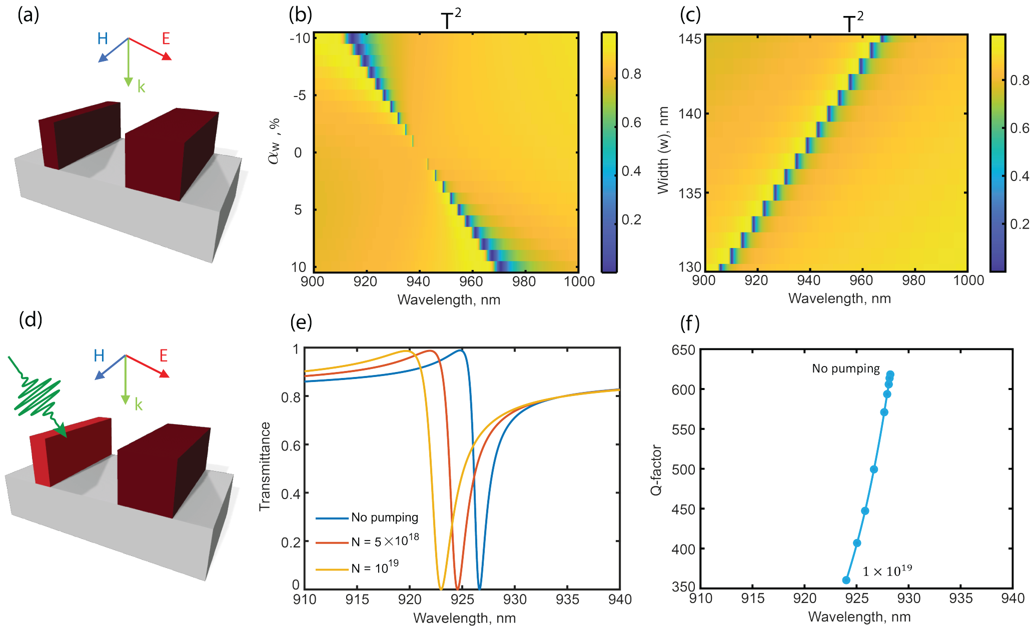

Fig. S4: (a) Designed nano-bar unit cell with a x-polarized probe light. (b) The transmittance spectrum of the with respect to width asymmetry α_w from -10% to 10%. (c) is the transmittance spectrum of different widths. (d) schematic of the unit cell with a left pumping pulse. The optical response is shown in (e) the transmittance spectrum with various free carrier densities and (e) the Q-factor of the resonance.

As we described in the main text, the aim of side pumping is to compensate for the asymmetry of width and permittivity. However, unlike the right bar pumping to "boost" the Q-factor of the resonance, we can pump the left bar to suppress its Q-factor, Figure S4(e). The resonance shows a blue shift, and the bandwidth becomes wider than the original state. Connecting with the Figure S4(f), the Q-factor at $\alpha_w = 5\%$ drop from 600 to 350. In the time delay spectrum, the resonance demonstrates an expansion behavior instead of squeezing.

S6 Model of resonance shift based on two-level carrier pumping

S6.1 Model function

Instead of using the step function to mimic permittivity change in the time domain, we fully modeled it from the two-level energy transition equation in the main text, Equation 4. By solving the equation, carrier density can be expressed as a function of time,

$$N(t) = C_1 \mathrm{e}^{-rt} - \frac{\mathrm{A}\,\tau_c \sqrt{\pi} \mathrm{e}^{\frac{r^2 \tau_c^2}{4}} \mathrm{e}^{-rt} \operatorname{erf}\left(\frac{r\tau_c}{2} - \frac{t}{\tau_c}\right)}{2}, \tag{S17}$$

where the r is the recombination rate of the GaAs material, the free carrier can live up to a nanosecond time scale, whose value is much larger than the decay of the mode. Therefore, this recombination effect

gives a less contribution to our following calculation. τ_c stands for the rising speed ratio of free carrier accumulation, described by the pumping Gaussian pulse.

By substituting the S17 into S11 and S12, we can obtain the refractive index and absorption change with the function of time at 930 nm. See following;

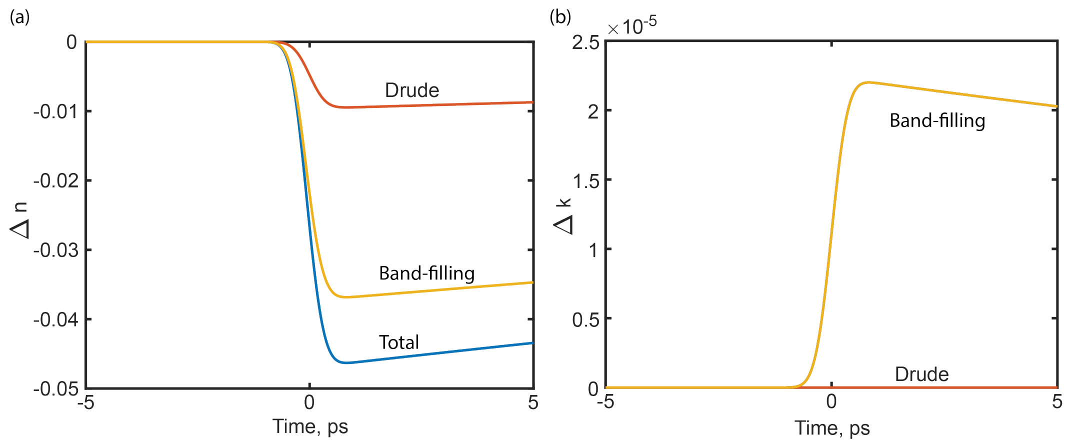

Fig. S5: (a) Refractive index and (b) extinction coefficient change of the GaAs as the function of time. The orange color is the domination of the Drude term, the yellow one is calculated for the band-filling term, and the blue curve is the total change.

According to the Figure, the Band-filling term plays a dominant role in both refractive index and extinction coefficient change. For Figure S5(b), the Drude term can be regarded as 0, and all impact of loss comes from the band-filling effect near the bandgap region. After that, we build the connection between the frequency shift of the mode and the permittivity change. By replacing the complex refractive index value with the permittivity, we can find the solution by equation S18,

$$\Delta\omega(t) = -\frac{\omega_0}{2}\frac{\int d^3\mathbf{r}\Delta\varepsilon(t)|\mathbf{E}(\mathbf{r})|^2}{\int d^3\mathbf{r}\varepsilon_0|\mathbf{E}(\mathbf{r})|^2} + O\left(\Delta\varepsilon(t)^2\right). \tag{S18}$$

In this equation, ω_0 ε are the original frequency and permittivity respectively. And $\mathbf{E}$ is the electric mode profile. Under the small perturbation, the error function, O, can be ignored, allowing us to treat the relationship between the change in permittivity and frequency as linear. Consequently, we establish a connection between time and frequency shifting, Shown in the main text Figure 4(a) orange solid line.

S6.2 Rising speed modeling

Although we established the connection between the time and frequency shifting, the related equation proves to be excessively complex, rendering direct implementation within the TCMT function challenging. To address this issue, we opt for utilizing the logistic function as it offers a more tractable curve-fitting approach compared to the amalgamation of multiple equations. The general equation is

$$f(x) = \frac{L}{1+e^{-p(x-x_0)}}., \tag{S19}$$

Here, the function $f(x)$ will be substituted with functions representing frequency and damping factor as a function of time. The variable p serves as a parameter to control the rising speed, analogous to the role of τ_c in Equation S17. Then, we obtained rising speed from $p = 1$ to $p = 100$ respectively.

According to the figure described above, the logistic function demonstrates an ultrafast shift in wavelength and damping factor with the variable time. By controlling the rising speed, we can observe

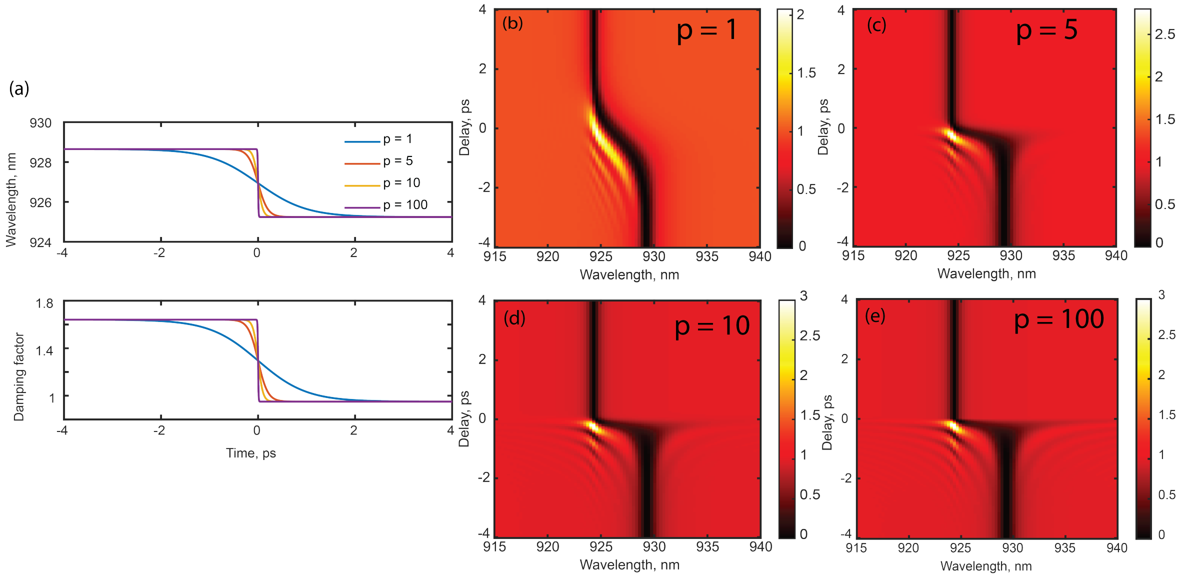

Fig. S6: (a) is the logistic function used in TCMT calculation to simplify the complexity. (b-e) demonstrate a transmittance spectrum from -4 to 4 ps time delay for different rising speed values p.

the optical response of the calculated two-dimensional TCMT transmittance spectrum. Interestingly, the interference enhancement at zero time delay is dependent on this rising speed, and it converges when the variable p is larger than 10^3, illustrated in the color bar of Figure S6(b-e). Therefore, the large transmittance enhancement at the near-zero time delay position, towards the wavelength shifting direction, can be obtained by providing large density and fast speed of free carrier injection.

S7 Numerical simulations: validation by FDTD

In this section, we demonstrate the transmittance spectrum, which has been simulated using FDTD, Lumerical. We positioned the central probe pulse in the time domain at $4ps$ and systematically varied the step function representing the permittivity change from 0 to $8ps$. All other configurations and settings remain consistent with those used for conventional metasurfaces. Then, we measure the transmittance spectrum, which showed a perfect match between the FDTD simulation and TCMT, particularly when the steepness of the logistic function (p) in TCMT is larger than 100, as illustrated in Figure S7.

References

[1] T. Weiss, M. Mesch, M. Schäferling, H. Giessen, W. Langbein, and E. A. Muljarov, "From dark to bright: first-order perturbation theory with analytical mode normalization for plasmonic nanoantenna arrays applied to refractive index sensing," *Physical review letters*, vol. 116, no. 23, p. 237401, 2016.

[2] K. Koshelev, S. Lepeshov, M. Liu, A. Bogdanov, and Y. Kivshar, "Asymmetric metasurfaces with high-q resonances governed by bound states in the continuum," *Physical Review Letters*, vol. 121, no. 19, p. 193903, 2018.

[3] R. Alaee, C. Rockstuhl, and I. Fernandez-Corbaton, "An electromagnetic multipole expansion beyond the long-wavelength approximation," *Optics Communications*, vol. 407, pp. 17–21, 2018.

[4] I. Fernandez-Corbaton, S. Nanz, R. Alaee, and C. Rockstuhl, "Exact dipolar moments of a localized electric current distribution," *Optics express*, vol. 23, no. 26, pp. 33044–33064, 2015.

[5] B. R. Bennett, R. A. Soref, and J. A. Del Alamo, "Carrier-induced change in refractive index of inp, gaas and ingaasp," *IEEE Journal of Quantum Electronics*, vol. 26, no. 1, pp. 113–122, 1990.

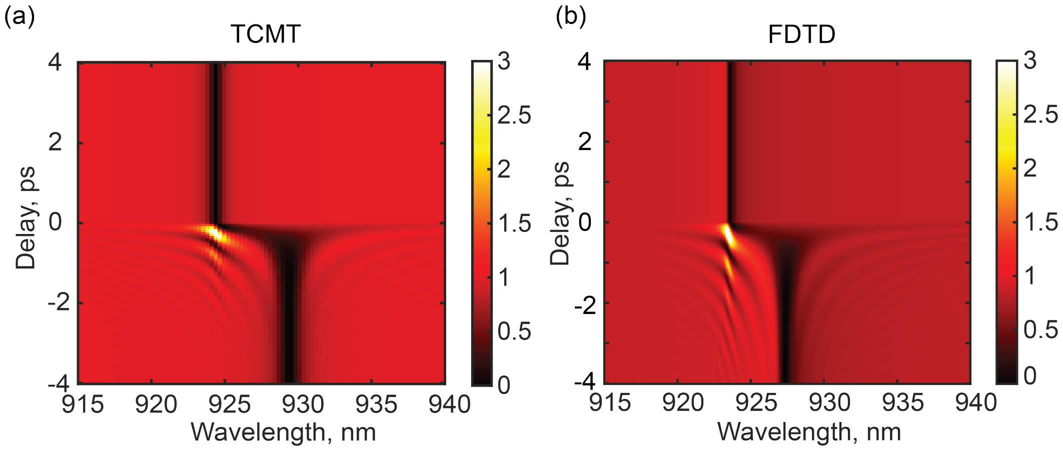

Fig. S7: (a) Transmittance spectrum with different delay times calculated by TCMT. (b) Transmittance spectrum with different delay times calculated by FDTD.

[6] B. Bennett and R. Soref, "Electrorefraction and electroabsorption in inp, gaas, gasb, inas, and insb," *IEEE journal of quantum electronics*, vol. 23, no. 12, pp. 2159–2166, 1987.

[7] M. R. Shcherbakov, S. Liu, V. V. Zubyuk, A. Vaskin, P. P. Vabishchevich, G. Keeler, T. Pertsch, T. V. Dolgova, I. Staude, I. Brener, *et al.*, "Ultrafast all-optical tuning of direct-gap semiconductor metasurfaces," *Nature Communications*, vol. 8, no. 1, pp. 1–6, 2017.

[8] C. Henry, R. Logan, and K. Bertness, "Spectral dependence of the change in refractive index due to carrier injection in gaas lasers," *Journal of Applied Physics*, vol. 52, no. 7, pp. 4457–4461, 1981.
